# Lipoproteins attenuate TLR2 and TLR4 activation by bacteria and bacterial ligands with differences in affinity and kinetics

**DOI:** 10.1186/s12865-016-0180-x

**Published:** 2016-10-28

**Authors:** Jeroen van Bergenhenegouwen, Aletta D. Kraneveld, Lieke Rutten, Johan Garssen, Arjan P. Vos, Anita Hartog

**Affiliations:** 1 Nutricia Research, Utrecht, The Netherlands; 2 Department of Pharmacology, Utrecht Institute for Pharmaceutical Sciences, Utrecht University, Utrecht, The Netherlands

## Abstract

**Background:**

The small intestine is a specialized compartment were close interactions take place between host, microbes, food antigens and dietary fatty acids. Dietary fats get absorbed by epithelial cells and processed into a range of lipoprotein particles after which they are basolaterally secreted and collected in the lymphatics. In contrast to the colon, the small intestine is covered only by a thin mucus coat that allows for intimate interactions between host-cells and microbes. Lipoproteins have long been recognized as protective factors in infectious diseases via the neutralization of bacterial toxins like lipopolysaccharides. Much less attention has been given to the potential role of lipoproteins as factors contributing to the maintenance of small intestinal immune homeostasis via modulating bacteria-induced immune responses.

**Results:**

Lipoproteins VLDL, LDL and HDL were found to neutralize TLR responses towards specific TLR-ligands or a selection of gram-negative and gram-positive bacteria. Attenuation of TLR2 activity was acute and only slightly improved by longer pre-incubation times of ligands and lipoproteins with no differences between bacterial-lipopeptides or bacteria. In contrast, attenuation of TLR4 responses was only observed after extensive preincubation of lipoproteins and LPS. Preincubation of bacteria and lipoproteins led only to a modest attenuation of TLR4 activity. Moreover, compared to TLR2, TLR4 activity could only be attenuated by lipoproteins over a small ligand dose range.

**Conclusions:**

These results demonstrate the ability of lipoproteins VLDL, LDL and HDL to inhibit TLR responses towards bacterial-ligands and bacteria. Presence of lipoproteins was found to modulate the MAMP-induced cytokine release by primary human monocytes measured as changes in the release of IL-6, TNFα, GM-CSF and IFNγ. Using TLR2 and TLR4-reporter cells, lipoproteins were found to inhibit TLR responses with differences in affinity and kinetics. These data establish a role for lipoproteins as immunoregulatory molecules, attenuating TLR-responses and thereby positively contributing to mucosal homeostasis.

**Electronic supplementary material:**

The online version of this article (doi:10.1186/s12865-016-0180-x) contains supplementary material, which is available to authorized users.

## Background

Apart from regulating lipid metabolism, evidence accumulates that lipoproteins are also involved in host-microbe interactions. It is well known that infection and inflammation induce an acute-phase response, leading to changes in plasma lipids and lipoprotein metabolism which in turn add to the inflammatory cycle potentially leading to atherosclerosis [1]. In addition, it has long been recognized that lipoproteins (i.e. chylomicrons, very low density lipoprotein (VLDL), low density lipoprotein (LDL) and high density lipoproteins (HDL)) interact with microbe associated molecular patterns (MAMP). Lipoproteins play an important role in the detoxification of MAMPs such as lipopolysaccharide (LPS) and lipoteichoic acid (LTA), most likely via sequestration of MAMPs, preventing Toll-like receptor (TLR) activation and the subsequent release of proinflammatory cytokines [2–6]. However, only a few studies report on the direct interaction of lipoproteins with bacteria and how this would affect subsequent recognition by TLRs. Studies in low density receptor deficient mice (LDLR^-/-^) mice, which suffer from increased circulating levels of LDL-cholesterol, showed that LDLR^-/-^ mice survive longer and have lower levels of circulating proinflammatory cytokine concentrations after infection with the Gram-negative bacteria *Salmonella typhimurium* and *Klebsiella pneumoniae* [7, 8]. Part of the protective effect of the increased circulating levels of LDL could be explained by the sequestration of LPS, but data indicated that the direct interaction of bacteria with lipoproteins might prohibit attachment of bacteria to host cells, preventing dissemination into the organs [8]. On the other hand, lipoprotein binding to bacteria might promote infectivity of host cells as has been shown for the intracellular human pathogens *Chlamydia pneumoniae* and *C. trachomatis* [9]. Overall, the data suggests that lipoprotein deposits on bacterial surfaces modulate host-microbe interactions.

Previous data indicates that preincubating cells with lipoproteins might potentiate subsequent TLR-responses, presumably via induction of Ca^2+^ mobilization [10]. Moreover, incubating cells with modified lipoproteins (i.e. oxidized LDL), which may be formed under inflammatory conditions, has been shown to induce TLR activity [11]. In this study, we analyzed the effects of lipoproteins on TLR-induced cellular activation by bacteria. To that end, TLR-ligands or bacteria were preincubated with lipoproteins before the addition of cells to rule out potential TLR-enhancing activities by lipoproteins. We further limit our studies to native lipoproteins to best mimic effects under homeostatic conditions. We show that VLDL, LDL and HDL attenuate TLR activity in response to Gram-positive and Gram-negative bacteria. TLR2-activity was attenuated immediately and over a larger dose-range compared to TLR4-activity, indicating differences in affinity and kinetics between lipoproteins, bacteria and TLR-induced cellular responses.

## Methods

### Bacterial fermentation and enumeration

*Lactobacillus salivarius* NutRes 283 and *Bifidobacterium breve* NutRes 200 were grown at 37 °C in a 400 ml reactor containing MRS broth (Oxoid, Badhoevedorp, The Netherlands) supplemented with 0.5 g/l L-cysteine for Bifidobacteria. The pH was maintained at 6.5 by addition of NaOH. To ensure anaerobic conditions the headspace was flushed with N_2_ or a gas mixture consisting of 5 % H_2_, 5 % CO_2_ and 90 % N_2_ for Bifidobacteria. Bacteria were harvested in the early stationary phase, washed in PBS and stored in glycerol 20 % (w/v), in aliquots at -80 °C. Cell counts were determined by plating serial dilutions (CFU) and fluorescent microscopy by staining with DAPI.

### Cell lines and Reagents

Cell viability reagent WST-1 was purchased from Roche Diagnostics, Almere, The Netherlands. Human serum and human plasma purified lipoproteins (VLDL, LDL, HDL) and apolipoproteins (ApoA, ApoB, ApoC1, ApoC2, ApoC3) were all purchased from Sigma-Aldrich. Human lipoprotein deficient serum was from Merck, Amsterdam, the Netherlands. Heat-killed *Staphylococcus aureus* (HKSA), *Escherichia coli* (HKEB), *Salmonella typhimurium* (HKST) were all purchased from Invivogen, Toulouse, France. Ultrapure LPS derived from *E. coli* K12 and purified LTA from *Staphylococcus aureus* (both from Invivogen, Toulouse, France) were used at the indicated concentrations. Synthetic bacterial lipopeptides Pam_3_CSK_4_, Pam_2_CSK_4_, FSL-1 (all from EMC microcollections, Tübingen, Germany) were used at the indicated concentrations. Non-phagocytic HEK293 TLR2-TLR6, HEK293 TLR4 stable transfectants and HEK293 TLR null control cells were purchased from Invivogen, Toulouse, France. HEK293 TLR2-TLR6 and HEK293 TLR null transfectants were stably transfected with the NFκB reporter plasmid pNiFty2-Luc, HEK293 TLR4 cells contained the NFκB reporter pNiFty2-SEAP (Invivogen, Toulouse, France). Cells were maintained in DMEM (Invitrogen) supplemented 10 % FBS, 4.5 g/L glucose and the appropriate antibiotics according to the manufacturer’s protocols.

### Primary monocytes

Human primary peripheral blood mononuclear cells (PBMCs) were isolated from buffy coats obtained from healthy blood donors at the Sanquin Bloodbank, Nijmegen, The Netherlands. The mononuclear cell fraction was obtained by density centrifugation of blood diluted 1:1 in PBS using Leucosep tubes (Greiner, Alphen a/d rijn, The Netherlands) according to the manufacturer’s instructions. Next, 1E+06 PBMCs in RPMI 1640 medium supplemented with 1 % heat inactivated fetal calf serum were seeded in 96-well flat bottom plates and allowed to adhere in a 5 % CO2 incubator at 37 °C. Non-adherent cells were removed and the adherent cells were washed 3 times with 37 °C RPMI 1640 medium. The adherent cell fraction was incubated with bacteria or bacterial ligands in stimulation assays as described below. Supernatants were collected after 16H and analyzed for the release of TNFα, interleukin-6, GM-CSF and interferonγ by multiplex detection immunoassays (Bio-Rad, veenendaal, The Netherlands). No cytokine release above detection level could be measured when primary monocytes were incubated with human serum or delipidated serum alone (data not shown).

### Stimulation assays

Bacteria or bacterial ligands were either directly seeded or pre-incubated, for 30 min unless otherwise indicated, into individual wells of a 96-wells plate with DMEM supplemented with 4.5 g/L glucose and 5 % human serum (HS), 5 % delipidated human serum (HSdelip) or 5 % HSdelip supplemented with 100 μg/ml of the lipoproteins (VLDL, LDL, HDL) or 5 μg/ml apolipoproteins (ApoA, ApoB, ApoC1, ApoC2, ApoC3) where indicated. After preincubation, 1E+05 TLR transfected cells were added to a final volume of 100 μl/well and incubated for 16H. The following day, HEK293 TLR2-TLR6 or HEK293 Null transfectants were analyzed for NFκB activation by measuring luciferase content via addition of 1 volume of the luciferase substrate: BriteLite (Perkin Elmer, Groningen, The Netherlands) after which Luminescence was measured. HEK293 TLR4 supernatants were analyzed for NFκB activity by measuring secreted embryonic alkaline phosphatase (SEAP) activity using QUANTI-Blue (Invivogen, Toulouse, France) after which OD was measured using a spectrophotometer. Bacteria were used at a ratio of 25:1 (bact:cell) unless otherwise indicated. TNFα was used at a concentration of 5 ng/ml. LPS was used at a concentration of 1 ng/ml and FSL-1 at 5 ng/ml unless otherwise indicated. None of the tested bacteria or bacterial ligands induced NFκB expression in the HEK293 TLR null control cells (data not shown). Cell viability was checked by adding WST-1 reagent to the cells, change in OD indicates cellular enzyme activity and functions as a measure for cell proliferation and viability.

### Statistical analysis

Statistical analyses were performed using the Student’s *t* test with the Graphpad Prism 6.02 statistical software. Differences were considered significant at *p* <0.05.

## Results

### TLR stimulation by bacteria or TLR-specific ligands is attenuated in the presence of serum lipids

**Fig. 1:**
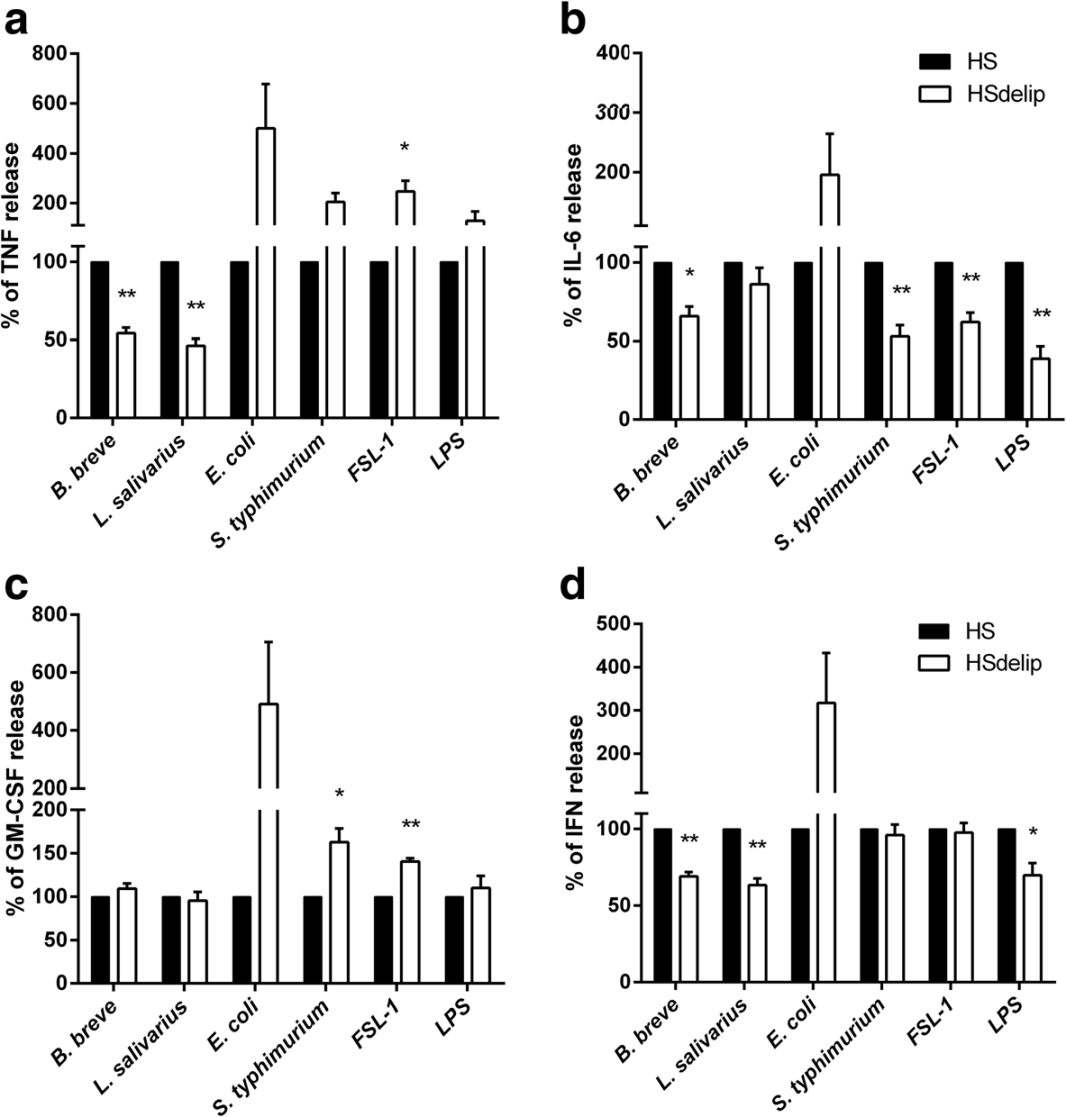
Serum lipoproteins modulate bacteria and bacterial-ligand induced immune responses. Primary monocytes were incubated with the intact bacteria *B. breve*,* L. salivarius*, *E. coli* and *S. typhimurium* at a ratio of 25:1 (bact:cell) or the bacterial ligands FSL-1 (100 ng/ml) or LPS (10 ng/ml). Supernatants were collected after 16H incubation and analyzed for the release of TNFα (**a**), IL-6 (**b**), GM-CSF (**c**) and IFNγ (**d**). Values represent mean ± SEM of 4 donors. Data is represented as percentage release compared to HS. Absolute cytokine levels are presented in Additional file 1. *: *p* < 0.05 **: *P* < 0.01

**Fig. 2:**
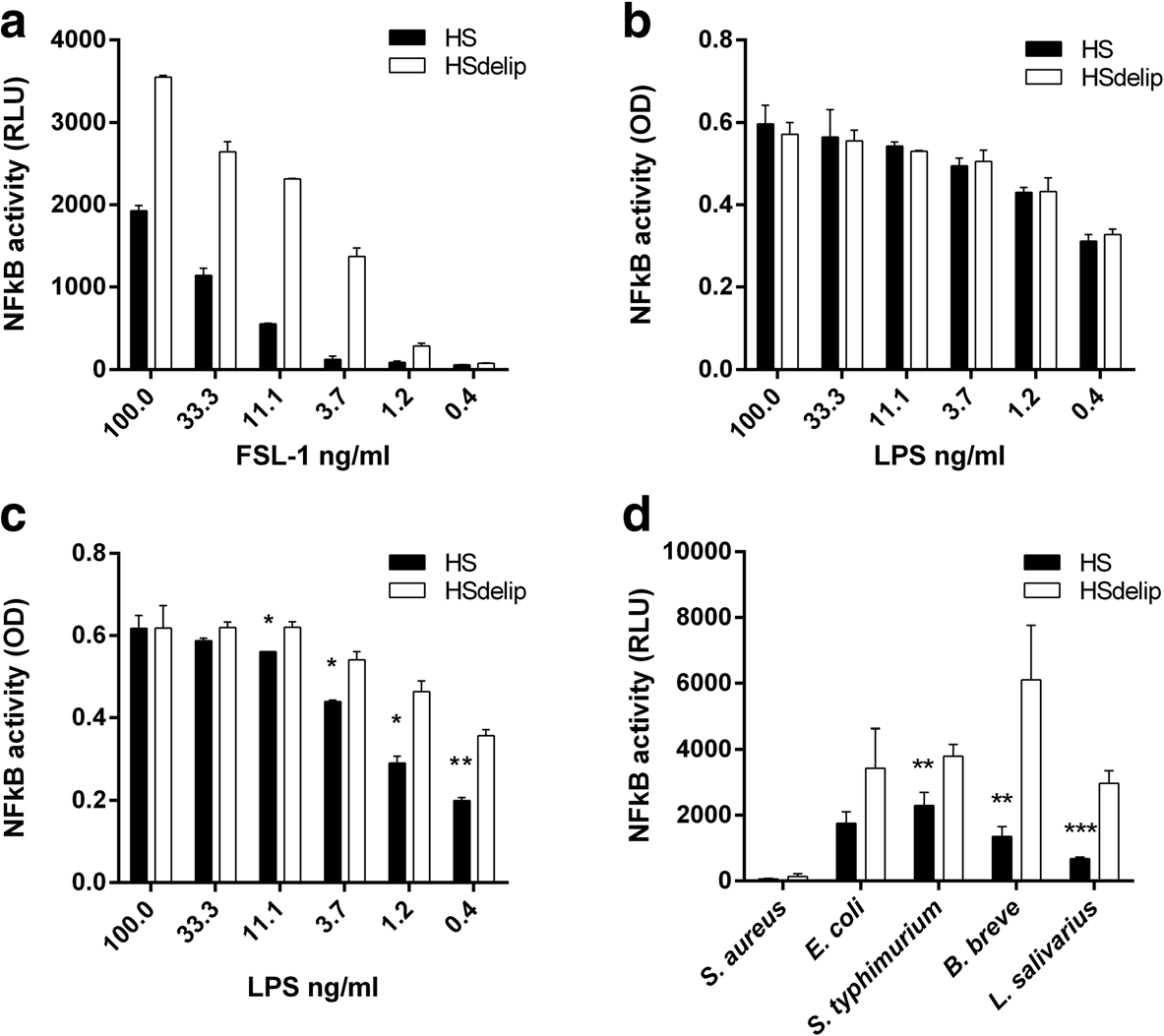
Effect of human serum and delipidated human serum on the ability of bacterial-ligands or bacteria to induce TLR2 or TLR4 activity. HEK-TLR2-TLR6 cells or HEK-TLR4 cells were incubated with a dose range of FSL-1 or LPS respectively in the presence of human serum (HS) or delipidated human serum (HSdelip). FSL-1 (**a**) but not LPS (**b**) activity was inhibited by HS compared to HSdelip. **c** LPS was preincubated with HS or HSdelip for 8 h before addition of HEK-TLR4 cells. LPS-activity was significantly inhibited by HS compared to HSdelip. **d** HEK-TLR2-TLR6 cells were incubated with *S. aureus*, *E. coli*, *S. thyphimurium*, *B. breve* or *L. salivarius* in the presence of HS or HSdelip. HS significantly inhibited *S. thyphimurium*, *B. breve* or *L. salivarius* induced TLR2/6 activity. Each value represents mean ± SD of triplicates. The experiment shown is representative of three separate experiments. *: *p* < 0.05 **:*P* < 0.01 ***:*P* < 0.001 determined by comparison with HSdelip

Serum lipoproteins are known to bind and neutralize bacterial ligands. However, not much is known about how serum lipids modulate immune responses following challenges with intact bacteria. To investigate whether serum lipoproteins are able to modify cellular responses to bacteria, we challenged human primary monocytes with the gram-positive bacteria *Bifidobacterium breve* and *Lactobacillus salivarius*, the gram-negative bacteria *Escherichia coli* and *Salmonella typhimurium* and the TLR2 specific ligand FSL-1 and TLR4 specific ligand LPS in the presence of intact human serum (HS) or human serum depleted for lipoproteins (HSdelip) and measured cytokine release (Fig. 1 and Additional file 1). Data indicates that TNFα, IL-6, GM-CSF and IFNγ release were affected by the presence of serum lipoproteins depending on bacterial strain or TLR-ligand. Cellular responses toward bacteria are for a large part determined by the activity of TLRs. To determine to what extend serum lipoproteins affect surface TLR activity we investigated the neutralization of LPS and a selection of TLR2 specific ligands (FSL-1, Pam_2_CSK_4_, Pam_3_CSK_4_ and LTA), by comparing the TLR activity in HS and HSdelip using TLR reporter cells. TLR2 activity in response to TLR2-specific ligands was significantly inhibited over a wide dose-range in the presence of HS compared to HSdelip (Fig. 2a and Additional file 2). To rule out any effect of the presence or absence of serum lipoproteins on cell growth we tested for changes in cell proliferation and viability as described in materials and methods. No changes in cellular conditions could be observed upon culture in HS or HSdelip (Additional file 2). TLR4 activity in response to LPS stimulation was not differently affected by the presence of either HS or HSdelip (Fig. 2b). Preincubating LPS for 8 h with HS or HSdelip, before the addition of TLR4 reporter cells, led to a attenuation of TLR4 activity observed from a dose of 11.1 ng/ml and lower (Fig. 2c). To investigate whether TLR2 activation following bacterial stimulation was similarly affected, we incubated TLR2 reporter cells with the intact bacteria *S. aureus*, *E. coli*, *S. typhimurium*, *B. breve* and *L. salivarius*. Presence of serum lipids attenuated TLR2 activity, suggesting a non-specific effect of serum lipids on bacterial induced TLR2 activation (Fig. 2d).

### VLDL, LDL and HDL serum lipid-fractions attenuate bacterial and ligand induced TLR activity

**Fig. 3:**
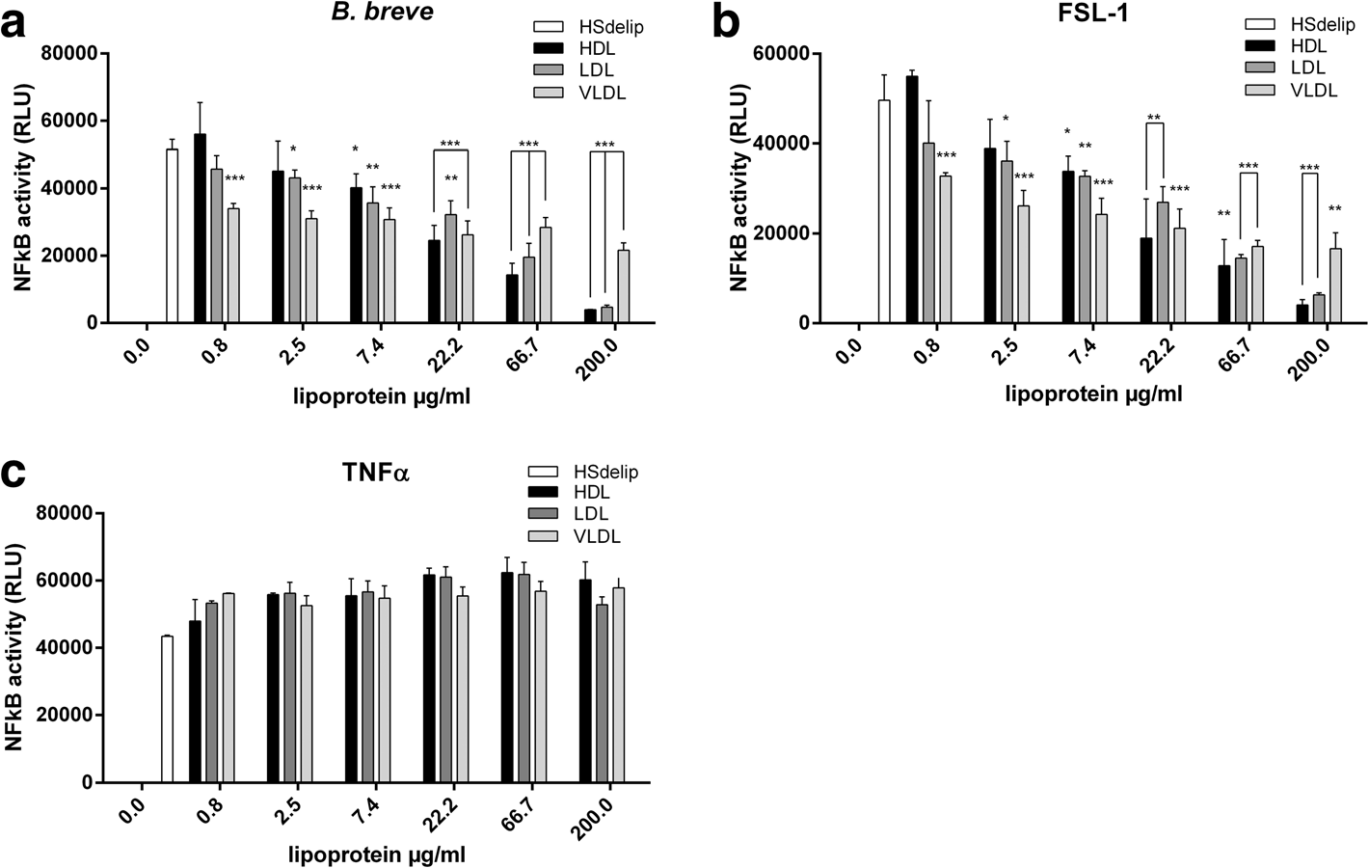
Effect of HSdelip or HSdelip supplemented with VLDL, LDL or HDL on TNFα, *B. breve* or FSL-1 induced NFκB activity. HEK-TLR2-TLR6 cells were incubated with *B. breve*, FSL-1 or TNFα in the presence of HSdelip or HSdelip supplemented with a dose range of the lipoprotein fractions HDL, LDL or VLDL. TLR2/6 activity in response to *B. breve* (**a**) or FSL-1 (**b**) was dose-dependently inhibited by the different lipoprotein fractions. **c** None of the lipoprotein fractions inhibited the TNFα induced NFκB activity. Each value represents mean ± SD of triplicates. The experiment shown is representative of three separate experiments. *: *p* < 0.05 **:*P* < 0.01 ***:*P* < 0.001 determined by comparison with HSdelip

**Fig. 4:**
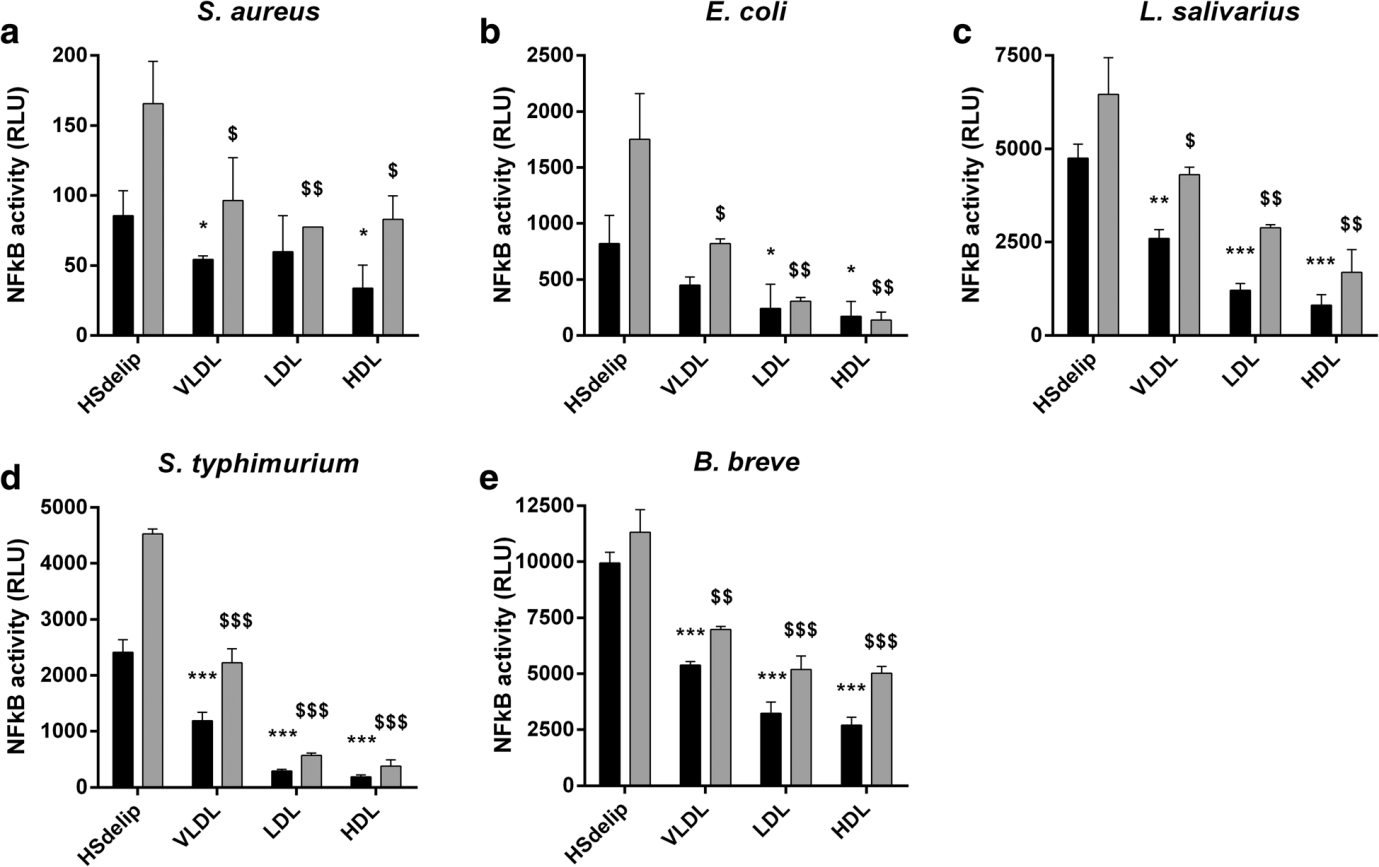
Effect of HSdelip or HSdelip supplemented with VLDL, LDL or HDL on bacteria-induced TLR2 activity*.* HEK-TLR2-TLR6 cells were incubated *with S. aureus (* ***a*** *), E. coli (* ***b*** *), L. salivarius (* ***c*** *)*, *S. typhimurium (* ***d*** *)* or *B. breve (* ***e*** *)* at a ratio of 10:1 (black bars) or 25:1 (grey bars) (bact:cell) in the presence of HS or HSdelip supplemented with the different lipoprotein fractions. HDL, LDL or VLDL significantly inhibited bacterial induced TLR2 activation. Each value represents mean ± SD of triplicates. The experiment shown is representative of three separate experiments. *:*p* < 0.05 **:*P* < 0.01 ***:*P* < 0.001 determined by comparison with HSdelip 10:1. ^**$**^:*p* < 0.05 ^**$$**^:*P* < 0.01 ^**$$$**^:*P* < 0.001 determined by comparison with HSdelip 25:1

**Fig. 5:**
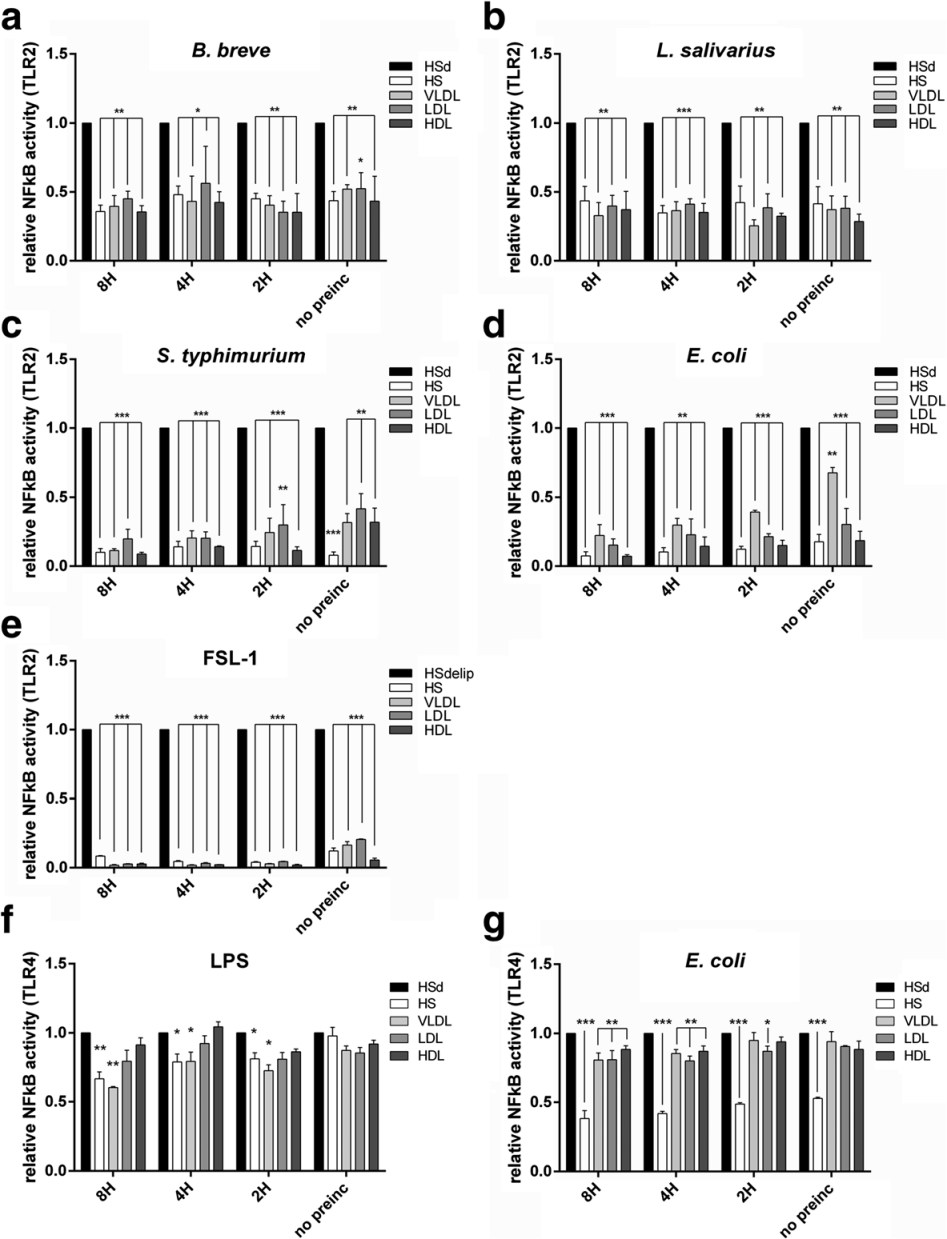
Effect of HS, HSdelip or HSdelip supplemented with VLDL, LDL or HDL on the ability of bacterial-ligands or bacteria to induce TLR2 or TLR4 activity. Bacteria or bacterial ligands were preincubated for the indicated times with either HS, HSdelip (HSd) or HSdelip supplemented with the separate lipoproteins before the addition of TLR-transfected cells. TLR2 activity in response to ligation by *B. breve* (**a**) and *L. salivarius* (**b**) was attenuated by HS or HSdelip supplemented with VLDL, LDL or HDL. No effect between the different preincubation times could be observed. TLR2 activity in response to ligation with *E. coli* (**c**), *S. thyphimurium* (**d**) or FSL-1 (**e**) was attenuated by HS or HSdelip supplemented with VLDL, LDL or HDL. This effect increased with prolonged preincubation. TLR4 activity in response to ligation with *E. coli* (**f**) or LPS (**g**) was attenuated by HS or HSdelip supplemented with VLDL, LDL or HDL. This effect increased with prolonged preincubation. Each value represents mean ± SD of triplicates. The experiment shown is representative of three separate experiments. *: *p* < 0.05 **:*P* < 0.01 ***:*P* < 0.001 determined by comparison with HSdelip

To identify the lipid fraction in human serum inducing the observed inhibitory effect on TLR2 activation, we stimulated TLR2 reporter cells with *B. breve* and FSL-1 in the presence of VLDL, LDL or HDL fractions. All lipid fractions attenuated TLR2 induced NFκB activity after stimulation with *B. breve* or FSL-1 (Fig. 3a,b). However, differences in effectiveness between lipoprotein fractions could be observed. VLDL was already effective at a low concentration which did not increase with increasing dose. In contrast, HDL and LDL clearly showed a dose-dependent effect. To investigate whether or not the observed effect was TLR specific we performed a similar experiment using stimulation with the cytokine TNFα. None of the lipid fractions were able to attenuate TNFα induced NFκB activity (Fig. 3c). To extend our findings to a selection of different bacteria, we incubated *S. aureus*, *S. typhimurium*, *E. coli*, *L. salivarius* and *B. breve* with TLR2 reporter cells in the presence of the different serum lipid fractions. Again, bacteria-induced TLR2 responses were attenuated in the presence of VLDL, LDL or HDL (Fig. 4). We next investigated whether prolonged preincubation of bacteria with the different lipoprotein fractions would even further attenuate TLR2 responses. In addition, we addressed lipid preincubation effects on TLR4 activity in response to LPS or the gram-negative bacteria *E. coli*. TLR2 activity after ligation by *B. breve* and *L. salivarius* was approximately 50 % reduced by the presence of HS or the different lipid-fractions compared to HSdelip while no differences could be observed between direct incubation or preincubation (Fig. 5a,b). TLR2 activity in response to *E. coli* and *S. typhimurium* without preincubation also showed approximately 50 % inhibition however, preincubation for up to 8 h further increased the inhibitory effect of the lipoprotein fractions most clearly observed in the VLDL fraction (Fig. 5c,d). FSL-1 induced TLR2 activity was approximately 75 % reduced by serum or lipoprotein fraction without preincubation, preincubation for 2 h or more led to an almost complete inhibition of TLR2 activity (Fig. 5e). TLR4 activity in response to ligation by LPS was found to decrease with preincubation time, reaching approximately 40 % reduction by HS and VLDL after 8 h of preincubation (Fig. 5f). Preincubation with LDL or HDL did not lead to a significant reduction of TLR4 activity. Intact serum inhibited TLR4 activity in response to *E. coli* by approximately 50 % with no preincubation till 60 % with 8 h of preincubation (Fig. 5g). A minimum of 2 h of preincubation of the separate lipoprotein fractions with *E. coli* was necessary to significantly reduce TLR4 activity. Overall, these results suggest a role for VLDL, LDL and HDL in the inhibition of TLR-induced cellular activation observed in the presence of human serum. Moreover, since non-TLR induced cellular activity was not affected, VLDL, LDL and HDL seem to specifically inhibit TLR-activity in response to bacterial and ligand stimulation.

### Apolipoproteins are not the main drivers of the TLR2 inhibitory effect

**Fig. 6:**
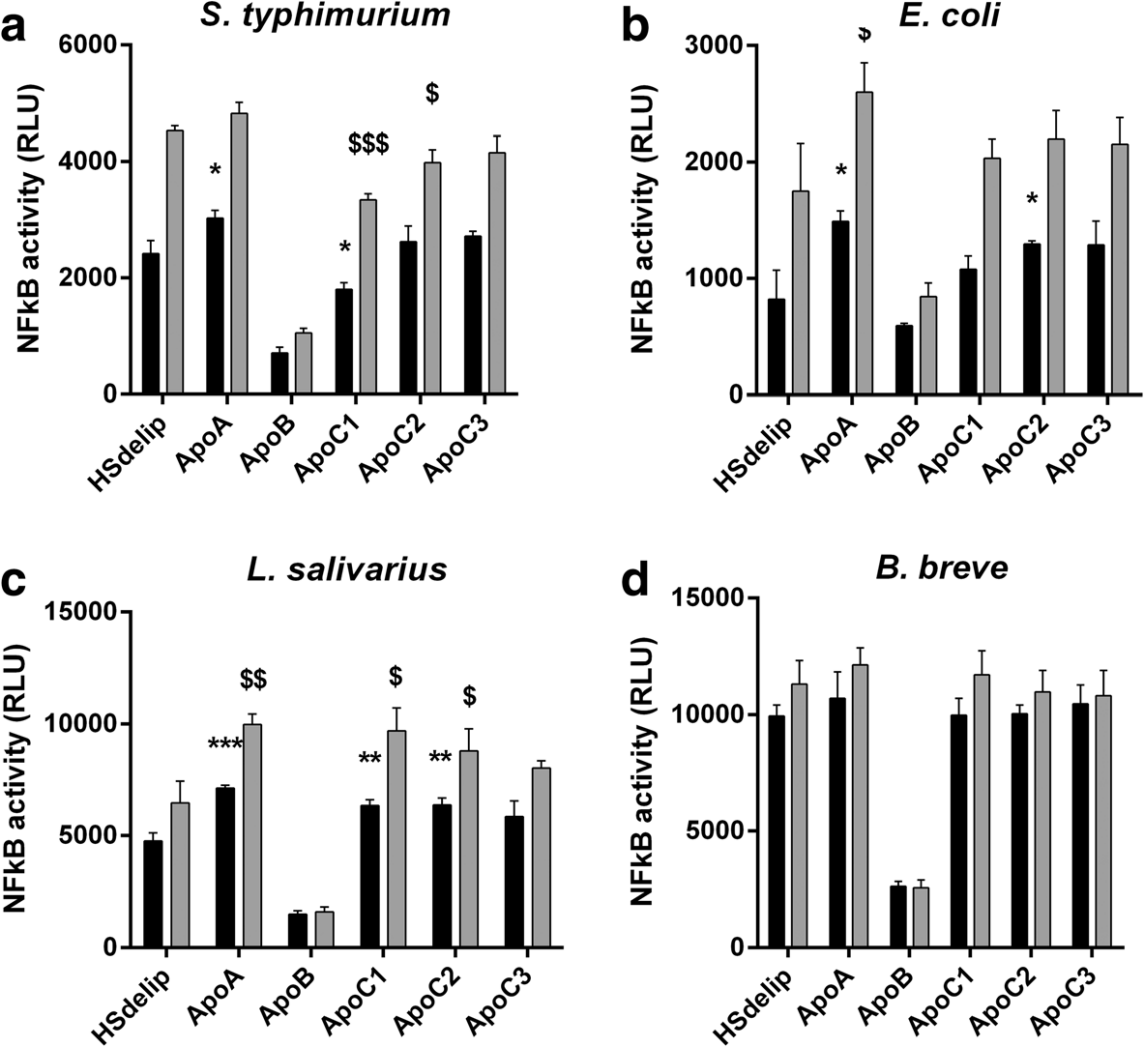
Effect of HSdelip or HSdelip supplemented with different apolipoproteins on the ability of bacteria to induce TLR2 activity. HEK-TLR2-TLR6 cells were incubated with *S. typhimurium, E. coli, L. salivarius* or *B. breve* at a ratio of 10:1 (black bars) or 25:1 (grey bars) (bact:cell) in the presence of HSdelip or HSdelip supplemented with the different apolipoproteins. ApoA enhanced, while ApoB and ApoC1 reduced, *S. typhimurium* (**a**) induced TLR2 activity, while ApoC2 and ApoC3 had no effect. ApoA and ApoC1-3, but not ApoB, were found to increase *E.coli* (**b**) or *L.salivarius* (**c**) induced TLR2 activity. No effect of ApoA and ApoC1-3 could be observed on *B. breve* (**d**) induced TLR2 activity, accept for ApoB which reduced TLR2 activity. Each value represents mean ± SD of triplicates. The experiment shown is representative of three separate experiments. . *:*p* < 0.05 **:*P* < 0.01 ***:*P* < 0.001 determined by comparison with HSdelip 10:1. ^**$**^:*p* < 0.05 ^**$$**^:*P* < 0.01 ^**$$$**^:*P* < 0.001 determined by comparison with HSdelip 25:1

Lipoproteins VLDL, LDL, and HDL contain apolipoproteins as structural components. Several classes and sub-classes exist which provide additional functions as cofactors for enzymes and ligands for cell-surface receptors [12, 13]. Previous data suggest that apolipoproteins have a role in the attenuation of TLR-signaling after ligation by bacterial lipoproteins [14]. To this end, we evaluated lipid-free apolipoprotein A, B, C1, C2 and C3 for their effect on bacterial induced TLR2 stimulation. Apolipoprotein A (ApoA) increased TLR2 activity against all strains tested accept for *B. breve*, in contrast to apolipoprotein B (ApoB) which inhibited TLR2 activity (Fig. 6). However, we observed that the inhibitory effect of ApoB was due to cellular toxicity (data not shown). Lipid-free ApoB, as used in our studies, was previously recognized to be toxic after addition to cell-cultures [15]. Apolipoprotein C1 (ApoC1), C2 (ApoC2) and C3 (ApoC3), showed a more strain dependent effect on TLR2 activity. ApoC1, but not ApoC2 nor ApoC3, slightly attenuated TLR2 activity in response to *S. typhimurium*. ApoC2 slightly enhanced TLR2 activity following stimulation with *S. typhimurium*, *E. coli* or *L. salivarius* while having no effect on stimulation by *B.breve*. ApoC3 slightly enhanced TLR2 activity in response to *S. typhimurium*, *E. coli* or *L. salivarius* but this did not reach significance. Overall, apolipoproteins by themselves are not the main drivers of the observed attenuation of TLR-activity due to serum lipids. However, our observations suggest that apolipoproteins play a role in bacterial induced TLR activity, but their contribution to the inhibitory actions of VLDL, LDL or HDL remains to be determined.

## Discussion

Cells of the small intestine perform many functions. For instance, small intestinal epithelial cells are critically important in the absorbance and processing of dairy fatty acids and a specialized subset of epithelial cells (microfold or M-cells) line specific compartments (Peyer’s patches) that are involved in luminal sampling. After processing of dietary fats into lipoproteins they are basolaterally collected in lymph ducts and move via the mesenteric lymph into the circulation as chylomicrons, VLDL, LDL or HDL particles [16]. Antigens are taken up by Peyer’s patch resident antigen presenting cells and move via the lymphatics to the mesenteric lymph node (MLN) were, in a normal healthy and unchallenged condition, tolerance is established [17, 18]. Microbes, either sampled from Peyer’s patches or directly via dendritic cell capture, are similarly carried to the MLN which acts as a firewall and prevents further dissemination [17, 19]. Compared to the colon, the small intestine is covered by a relatively thin mucus-layer that enables direct and frequent interactions between commensals, probiotics and food-borne pathogens and mucosal immune cells [20]. Therefore, the small intestine can be seen as a compartment were constant interactions take place between antigens (food, bacterial) and lipoproteins. Although much is known about the interaction of lipoproteins with bacterial ligands, not much is known on how lipoproteins interact with intact bacteria and how this would affect subsequent immune responses.

Here, we show that the presence of lipoproteins inhibit TLR-activation in response to both specific TLR-ligands as well as a broad selection of gram-negative as well as gram-positive bacteria. We have shown that specific TLR-ligands interact with lipoproteins with differences in kinetics and affinity. For lipoproteins to inhibit TLR4 activation by LPS, extensive preincubation of LPS with lipoproteins before the addition of cells was needed. This is in agreement with previous data where preincubation for at least 4 h was necessary to neutralize LPS. Moreover, similar to our findings, neutralization of LPS was maximal at dosages below 10 ng/ml [21]. Previous published data demonstrated that serum lipoproteins are able to attenuate TLR2-induced macrophage activation in response to both purified LTA from *Staphylococcus aureus* and recombinant bacterial lipopeptides, mimicking cell wall fragments from *Chlamydia trachomatis* and *Borrelia burgdorferi*. Similar to our observations, lipoprotein neutralization of the bacterial products was accomplished without extensive preincubation [2, 14]. In the present study, we extended our observations to include intact bacteria. Bacteria-induced TLR2 activity was attenuated in the presence of intact serum, in contrast to delipidated serum, suggesting a role for lipoproteins. The molecular mechanisms of this process remain largely unknown. Lipoproteins offer valuable substrates for cellular growth and therefore a lack of lipoproteins might impact cell proliferation or subsequent cellular responses. However, no changes in cell viability or activity upon culture of the TLR-transfectants in the presence or absence of lipoproteins could be observed (Additional file 2). Moreover, as indicated by the stimulation with TNFα, non-TLR induced responses were not inhibited by the presence of lipoproteins suggesting the effects are limited to TLR-induced responses (Fig. 3). Taken together, these observations suggest that lipoproteins affect cellular responses via interference with ligand-TLR binding. Lipoprotein particles bear no TLRs on their surface, so interactions between bacterial ligands and lipoproteins are not governed by ligand-receptor interactions. Rather, bacterial ligands are thought to simply dissolve into the phospholipid coat of the lipoprotein, sequestering the lipid part of the ligand from insertion into the ligand-binding portion of TLRs [3]. All lipoproteins are made up of protein, phospholipids, cholesterol and triglycerides. However, only the phospholipid content correlates to the effectiveness of ligand neutralization [22]. Bacterial lipopeptides, in contrast to LPS, share structural similarities with phospholipids. Lipopeptides have a cysteine group attached to a glycerol subunit, while phospholipids have a phosphate group attached to a glycerol subunit. In both cases two fatty acyl chains are coupled to the glycerol subunits. We presume that the differences in molecular make-up, with regard to the number and make-up of fatty-acyl chains, between LPS and LTA or lipopeptides might therefore explain the difference in neutralization kinetics by lipoproteins. However, we could find no data substantiating this hypothesis. It is known that the plasma proteins soluble CD14 and LPS-binding protein (LBP) greatly facilitate LPS and LTA neutralization by lipoproteins [2, 23, 24]. In addition to LPS, LBP is reported to bind LTA as well as di and tri-acylated lipopeptides [25]. Since our TLR-transfected HEK cells constitutively express CD14, presence or absence of LBP does not explain the difference in kinetics between TLR2 and TLR4. However, since LBP in the circulation is found attached to lipoproteins [26], absence of LBP in delipidated HS or the different purified lipoprotein fractions might account for the differences between HS and HSdelip regarding their inhibitory effect on TLR4-activity in response to *E. coli*. Moreover, presence of LBP might be more crucial in neutralization of LPS compared to di or tri-acylated bacterial lipoproteins [14, 23]. Potentially, to compensate for the lower kinetics in neutralization of TLR4 ligands, the small intestine also locally produces LBP and the apolipoprotein serum amyloid A (SAA) that is known to contribute to the neutralization of gram-negative bacteria [27, 28]. Not much is known about how lipoproteins interact with intact bacteria. Bacterial cell-wall constituents like LPS, LTA and lipopeptides are carbohydrates or proteins bound to a lipid tail which is buried into the cell wall. It is therefore unlikely that the same principles that govern the interaction between lipoproteins and bacterial fragments equally apply to the interaction with intact bacteria. Lipid-free apolipoproteins play a role in bacteria-TLR interactions with the presence of apolipoproteins increasing TLR activity (Fig. 6). However, these findings are in apparent contrast to work done by Bas et al, where it was shown that apolipoproteins attenuate TLR-activity in response to bacterial lipopeptides [14]. These two findings may, at first sight, seem contradictory. However, interactions between the hydrophobic nature of apolipoproteins and the hydrophic part of bacterial lipoproteins could be envisaged leading on the one hand to sequestering of bacterial products and inhibition of TLR-activity, while on the other hand to deposition on the bacterial cell wall, acting as ligands for scavenger receptors that recognize apolipoproteins subsequently facilitating interaction with TLRs [29–31]. In support of this hypothesis, lysine residues of apoA were found to interact with bacterial cell walls based on electrostatic forces leading to deposition of apoA on the bacterial surface [32]. Moreover, the silkworm apoB homologue, apolipophorin, specifically interacts with LTA expressed on the bacterial cell surface [33, 34]. In addition, specific peptides derived from apoE were shown to have anti-microbial properties most likely via binding to LPS [35]. Overall, this indicates that lipoproteins interact with bacterial surfaces, through their apolipoprotein content, either via electrostatic interactions or by binding to specific ligands. Interestingly, mice deficient for either apoA or apoE show differences in their microbiota composition compared to wild type mice [36, 37]. However, it remains to be determined whether this is due to differences in the direct interactions between apolipoproteins and the microbiota or more indirectly through changes in host metabolism which may impact microbiota composition.

## Conclusions

Although lipoproteins are recognized as factors that play a role in innate immunity, much of the research efforts so far have focused on the role of circulating lipids and infection [3, 5]. We have provided data indicating that lipoproteins may also play a role in the maintenance of intestinal homeostasis by neutralizing potential harmful bacterial-derived ligands, as well as by modulating cellular responses towards bacteria. Using a selection of bacteria encompassing both gram-positive and gram-negative bacteria as a model for non-pathogenic encounter of host-cells with bacteria, lipoproteins were found to down modulate subsequent TLR responses to both gram-negative as well as gram-positive bacteria, a process recognized to be important for intestinal tolerance [38, 39]. These findings are relevant for our understanding of the immune response towards commensals and (potential) pathogens. Furthermore, these results may be important to consider when studying the effects of probiotic applications, e.g. in relation to the food matrix or dietary context in which the bacteria are supplied in or when translating effects from the in vitro to the in vivo situation.

## Additional files

Additional file 1: Serum lipoproteins modulate bacteria and bacterial-ligand induced immune responses. Primary monocytes were incubated with the intact bacteria B. breve, L. salivarius, E. coli and S. typhimurium at a ratio of 25:1 (bact:cell) or the bacterial ligands FSL-1 (100 ng/ml) or LPS (10 ng/ml). Supernatants were collected after 16H incubation and analyzed for the release of TNFα (A), IL-6 (B), GM-CSF (C) and IFNγ (D). Values represent mean ± SEM of 4 donors. (TIF 276 kb)
Additional file 2: Effect of HS or HSdelip on ligand induced TLR activity. HEK-TLR2-TLR6 cells were incubated with a dose range of (A)Pam_3_CSK_4_, (B) Pam_2_CSK_4_ or (C) LTA respectively in the presence of human serum (HS) or delipidated human serum (HSdelip). HS significantly inhibited ligand-induced TLR2 activity when compared to HSdelip. Effect of HS or HSdelip on cellular activity. HEK-TLR2-TLR6 cells were incubated in either HS or HSdelip. After overnight incubation 10 μl of the WST-1 reagent was added. (D) Change in OD was recorded over the indicated time points (minutes). No difference in cellular activity could be observed between HS or HSdelip. (TIF 305 kb)

## Abbreviations

Apo: Apolipoprotein
HDL: High density lipoproteins
LBP: LPS-binding protein
LDL: Low density lipoprotein
LPS: Lipopolysaccharide
LTA: Lipoteichoic acid
MAMP: Microbe-associated molecular pattern
MLN: Mesenteric lymph node
TLR: Toll-Like receptor
VLDL: Very low density lipoprotein
