# Supplementary figures and images for: Lipoproteins attenuate TLR2 and TLR4 activation by bacteria and bacterial ligands with differences in affinity and kinetics
Source: BMC Immunol. 2016 Oct 28;17:42. doi: 10.1186/s12865-016-0180-x (PMC5086051; doi:10.1186/s12865-016-0180-x)

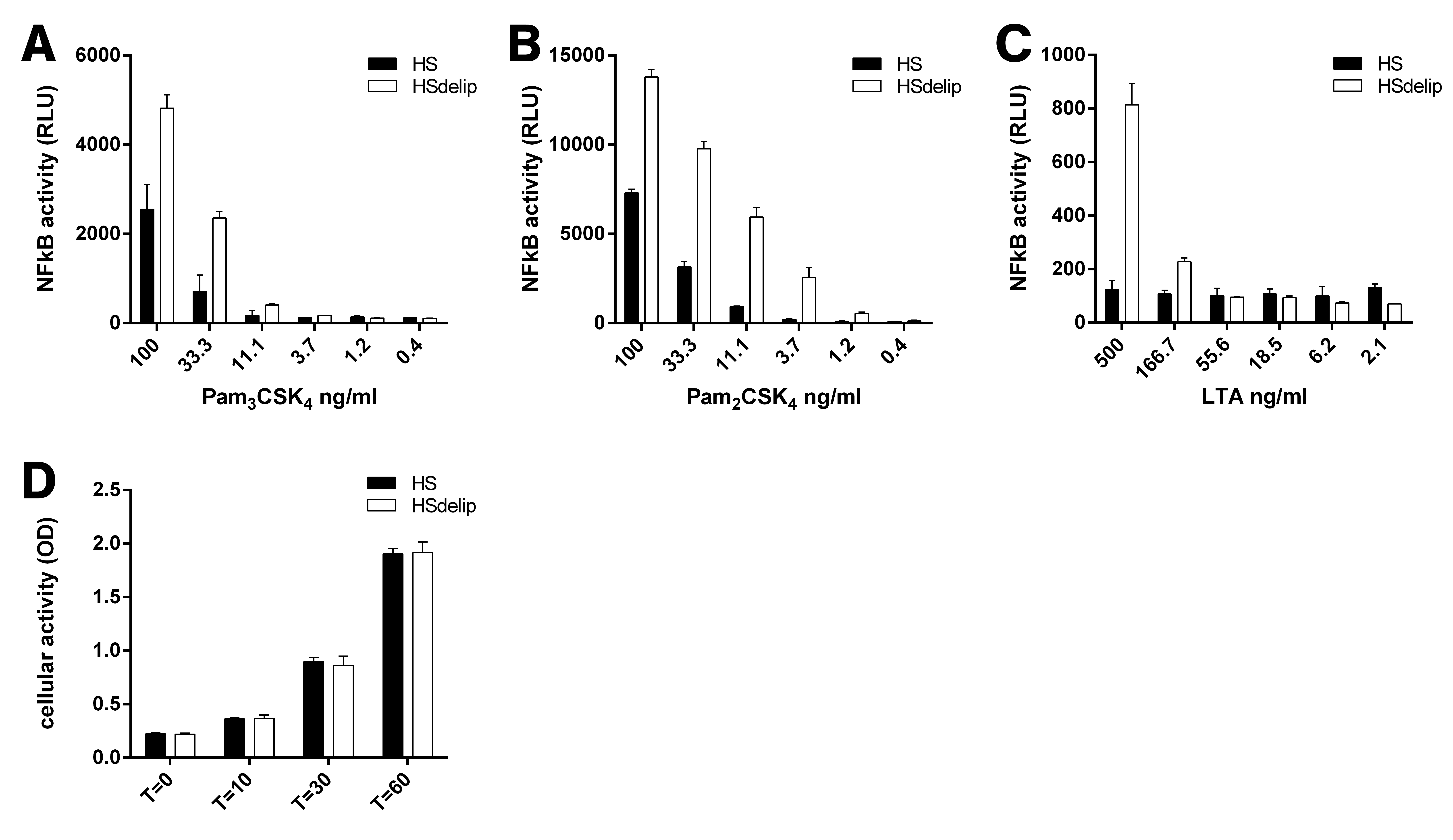

Supplement: Additional file 2: — Effect of HS or HSdelip on ligand induced TLR activity. HEK-TLR2-TLR6 cells were incubated with a dose range of (A)Pam3CSK4, (B) Pam2CSK4 or (C) LTA respectively in the presence of human serum (HS) or delipidated human serum (HSdelip). HS significantly inhibited ligand-induced TLR2 activity when compared to HSdelip. Effect of HS or HSdelip on cellular activity. HEK-TLR2-TLR6 cells were incubated in either HS or HSdelip. After overnight incubation 10 μl of the WST-1 reagent was added. (D) Change in OD was recorded over the indicated time points (minutes). No difference in cellular activity could be observed between HS or HSdelip. (TIF 305 kb) [file 12865_2016_180_MOESM2_ESM.tif]
